# High-Level Expression of Endo-β-N-Acetylglucosaminidase H from *Streptomyces plicatus* in *Pichia pastoris* and Its Application for the Deglycosylation of Glycoproteins

**DOI:** 10.1371/journal.pone.0120458

**Published:** 2015-03-17

**Authors:** Fei Wang, Xiaojuan Wang, Xiaolan Yu, Lin Fu, Yunyun Liu, Lixin Ma, Chao Zhai

**Affiliations:** Hubei Collaborative Innovation Center for Green Transformation of Bio-resources, Hubei Key Laboratory of Industrial Biotechnology, College of Life Sciences, Hubei University, Wuhan, People’s Republic of China; Russian Academy of Sciences, Institute for Biological Instrumentation, RUSSIAN FEDERATION

## Abstract

Endo-β-N-acetylglucosaminidase H (Endo H, EC3.2.1.96) is a glycohydrolase that is widely used in the study of glycoproteins. The present study aimed to assess the effect of high-level endo-β-N-acetylglucosaminidase H expression in *Pichia pastoris*. The DNA coding sequence of this enzyme was optimized based on the codon usage bias of *Pichia pastoris* and synthesized through overlapping PCR. This novel gene was cloned into a pHBM905A vector and introduced into Pichia pastoris GS115 for secretary expression. The yield of the target protein reached approximately 397 mg/l after a 6-d induction with 1% (v/v) methanol in shake flasks, which is much higher than that observed upon heterologous expression in *Escherichia coli* and silkworm. This recombinant enzyme was purified and its enzymatic features were studied. Its specific activity was 461573 U/mg. Its optimum pH and temperature were pH 5.5 and 37°C, respectively. Moreover, our study showed that the N-linked glycan side-chains of several recombinant proteins expressed in *Pichia pastoris* can be efficiently removed through either the co-fermentation of this recombinant strain with strains expressing substrates or by mixing the cell culture supernatants of the endo-β-N-acetylglucosaminidase H expressing strain with strains expressing substrates after fermentation. This is the first report of high-level endo-β-N-acetylglucosaminidase H expression in *Pichia pastoris* and the application of this enzyme in the deglycosylation of raw glycoproteins heterologously expressed in *Pichia pastoris* using simplified methods.

## Introduction

Endo-β-N-acetylglucosaminidase H (Endo H, EC3.2.1.96) is a glycohydrolase that is secreted by *Streptomyces plicatus* and a few other *Streptomyces* species [1]. It cleaves the β-1, 4-glycosidic bond of the N-acetyl glucosamine core of oligosaccharides and leaves one N-acetylchitobiose attached to the asparagine residue of the glycoprotein [2–3]. It can only remove the hybrid-type and high mannose-type N-glycan side-chains [4]. Therefore, Endo H is a powerful tool for investigating the functions and structures of glycoproteins and their precursors. The sensitivity to Endo H is commonly used to identify the composition of the glycan portion of N-glycosylated proteins and their moieties [5–8]. In addition, Endo H is also used industrially to remove glycan side-chains from recombinant proteins. More and more recombinant proteins are being generated using eukaryotic expression systems [9–10]. These products have been widely applied in industry and research due to their high yields and low costs. However, the post-translational modification system of eukaryotic cells may lead to the aberrant glycosylation of target proteins, which in turn may cause them to malfunction. Therefore, glycohydrolases such as glycopeptidase F (PNGase F) and Endo H may be used to remove N-glycan side-chains from glycoproteins [11–12].

Commercial Endo H can be extracted directly from *Streptomyces plicatus* or obtained via heterologous expression in *E*. *coli*. Robbins et al. previously expressed Endo H in *E*. *coli* using the P_L_ promoter of λ phage, leading to the secretion of the recombinant protein into the periplasmic space. The Endo H expression level increased by approximately 150-fold in 90 min at 42°C compared with its endogenous expression level. Approximately 23 mg (6000 U) of recombinant protein could be purified from 4 l of cell culture[13–14]. Endo H was also secretory expressed in silkworm using silkworm- baculovirus expression system. About 30 μg Endo H was recovered from 10 ml silkworm hemolymph and the expression level was 3 mg/l. And the same paper mentioned that the activity in silkworm was comparable with *E*. *coli* [15]. In the present study, we report the secretory expression of Endo H in *P*. *pastoris* for the first time. The Endo H expressed in this study was about 397 mg/l. Moreover, we attempted to use recombinant Endo H to deglycosylate the recombinant proteins expressed and modified by *P*. *pastoris* through both co-fermentation and post-fermentation treatments. The N-linked glycan side-chains of the target proteins could be efficiently removed using both methods, indicating that the genetically engineered strain constructed in this study can be utilized for the convenient preparation of non-glycosylated recombinant proteins.

## Materials and Methods

### Strains, vectors, media and reagents

*E*. *coli* DH10β and *P*. *pastoris* GS115 were obtained from Invitrogen (USA). The pMD18-T vector was purchased from Takara (JPN). The yeast expression vector pHBM905A (*ColE1 ori*, *Amp* ^*r*^, *Kan* ^*r*^, *HIS4*, *P*_*AOX1*_, *T*_*AOX1*_) was stored in our laboratory [16]. Luria-Bertani (LB) medium was prepared for the cultivation of *E*. *coli* as described in the Manual of Molecular Cloning [17]. For yeast culture, Buffered glycerol-complex medium (BMGY), Buffered methanol-complex medium (BMMY) and minimal dextrose medium (MD) were prepared according to the *Pichia* Expression Kit (Invitrogen, USA). RNase B, commercial Endo H and PNGase F were purchased from NEB (USA). Pfu DNA polymerase and Pierce Glycoprotein Staining Kit were from Thermo Scientific (USA). Carboxypeptidase Y from baker's yeast (CPY) was from Sigma (USA). Human erythropoietin (EPO) expressed in CHO cells was from Cusabio (China). All other reagents used in this study were of analytical grade.

### Design and synthesis of the *Endo H-P* gene

The DNA coding sequence of *Endo H* from *S*. *plicatus* was optimized to match the codon usage preference of *P*. *pastoris*. To synthesize this modified ORF, 26 oligonucleotides were designed for overlapping PCR using the DNAWorks program (http://helixweb.nih.gov/dnaworks) (Table 1). The gene synthesis procedure was carried as described previously [18]. The oligonucleotides encoding both ends of the gene, EndoH-1 (0.2 μmol), EndoH-26 (0.2 μmol) were mixed with lower amount of the rest oligonucleotides (0.03 μmol) and PCR was carried out under following condition (25 cycles): 98°C, 30 s for denaturing; 58°C, 30 s for annealing; 72°C, 50 s for elongation, followed by extension at 72°C for 7 min. MutS (mismatch-binding protein) (2.5 μg) was used to treat 50 ng PCR products in order to reduce base mismatches, short deletions and insertions produced during the process of PCR[19]. The process of PCR and MutS treatment was repeated twice. The synthesized DNA fragment was inserted into pMD18-T and transformed into *E*. *coli* DH10β. Proper construction of recombinant plasmid was confirmed by DNA sequencing (Sangon, China).

**Table 1:** Primers used for overlapping PCR in this study.

Primers	Oligonucleotide Sequences (5'→3')
EndoH-1	Gtcagctccagctccagttaagc
EndoH-2	Tattaacttcaacgtaagcaacggaagttggaccttgcttaactggagctggagc
EndoH-3	Ccgttgcttacgttgaagttaataacaactctatgttgaatgttggtaagtacac
EndoH-4	Tcaaaagcgttaccaccgccatcagccaaagtgtacttaccaacattcaacatag
EndoH-5	Gcggtggtaacgcttttgatgtggctgttatattcgctgctaacattaactacga
EndoH-6	Ttaaaatgcaagtaagcagtcttagtaccagtatcgtagttaatgttagcagcga
EndoH-7	Ctaagactgcttacttgcattttaacgaaaacgttcagagagttttggataacgc
EndoH-8	Cttgttgttgcaatggtctaatttgagtaacagcgttatccaaaactctctgaac
EndoH-9	Aaattagaccattgcaacaacaaggtattaaggttttgttgtctgttctaggcaa
EndoH-10	Atgggaagttagcgaaaccggcaccttgatggttgcctagaacagacaacaaaac
EndoH-11	Cggtttcgctaacttcccatctcaacaagctgcttctgcttttgccaagcaattg
EndoH-12	Ctccatccagaccgtacttagcgacggcatcagacaattgcttggcaaaagcaga
EndoH-13	Ctaagtacggtctggatggagttgatttcgatgatgaatacgctgaatatggtaa
EndoH-14	Gaagaatcgtttggttgagcagtaccgttgttaccatattcagcgtattcatcati
EndoH-15	Tgctcaaccaaacgattcttcttttgttcatttggttactgctttgagagctaat
EndoH-16	Atattgtatagagaaataatcttatctggcatattagctctcaaagcagtaacca
EndoH-17	Gccagataagattatttctctatacaatataggtccagctgccagtaggctatct
EndoH-18	Gtaatcaaacttatcagaaacatcaacgcctccgtaagatagcctactggcagct
EndoH-19	Gttgatgtttctgataagtttgattacgcttggaacccatactacggaacttggc
EndoH-20	Aagctgagcctttggcaaggcaatacctggaacctgccaagttccgtagtatggg
EndoH-21	Cttgccaaaggctcagcttagtccagcagctgttgaaattggcagaacttctcgt
EndoH-22	Caacagttcttctagccaaatcagcaacagtagaacgagaagttctgccaatttc
EndoH-23	Tgatttggctagaagaactgttgatgaaggttacggtgtttatctaacttacaac
EndoH-24	Acgtcagcagttctatcaccaccgtccaggttgtaagttagataaacaccgtaac
EndoH-25	Ggtgatagaactgctgacgtttctgcctttactagagaattgtacggttctgaag
EndoH-26	ggccattatggagttctaacagcttcagaaccgtacaattctctag

Note: The underlined EndoH-1 and EndoH-26 sequences matched the sticky ends generated by *Cpo*I and *Not*I, respectively.

### Constructing the expression vector for the recombinant expression of *Endo H* in *P*. *pastoris* GS115

The target gene was amplified from the recombinant T-vector using the primers EndoH-1 and EndoH-26 (Table 1). The resulting PCR product was treated with T4 DNA polymerase at 12°C for 20 min in the presence of 1 mM dTTP and ligated into a pHBM905A vector digested with *Not*I and *Cpo*I [16]. Recombinant plasmids were identified by DNA sequencing and linearized using *Sal*I, followed by transforming into *P*. *pastoris* GS115 via electroporation (4 kΩ, 50μF, 400 V). Transformants were screened on MD plates containing 0.4 mg/l biotin without histidine and identified by PCR with primer pair EndoH-1 and EndoH-26.

### Expression of recombinant Endo H in *P*. *pastoris* GS115

Recombinant *P*. *pastoris* GS115 bearing the target gene in its genome was incubated in 100 ml of BMGY until the OD_600_ value reached approximately 20. All cells were harvested by centrifugation and transferred to 25 ml BMMY. Two hundred and fifty microliter of 1% (v/v) methanol was added every 24 h to induce target protein expression. Approximately 1 ml of cell culture was collected every 24 h and centrifuged at 6,000×g for 5 min to remove cells. After 144 h, an equal volume of each supernatant (15 μl) was loaded onto a 12% (w/v) polyacrylamide gel for SDS-PAGE, followed by staining with Coomassie Brilliant Blue G-250. The total protein concentration in each supernatant was measured using the Micro-BCA Protein Assay Reagent (Pierce, USA).

### Purification of the recombinant Endo H

After induction with methanol for 120 h, the target protein was purified by centrifuging approximately 20 ml of BMMY cell culture at 6000×g for 5 min. The supernatant was then collected and dialyzed with a Millipore 10-kDa cut-off membrane at 4°C to remove ions and salts. The sample was resuspended in 15 ml of Buffer A (20 mM Tris-HCl; 5 mM Na_2_EDTA; pH 7.5). The sample was centrifuged at 5000×g to the final volume of 1 ml. This step was repeated twice. The 1-ml solution was added to the diethylaminoethyl dextran gel (DEAE) FF and washed under the following condition: Buffer A (20 mM Tris-HCl; 5 mM Na_2_EDTA; pH 7.5); Buffer B (20 mM Tris-HCl; 2M NaCl; 5 mM Na_2_EDTA; pH 7.5), with the velocity of 0.5 ml/min. The target protein was eluted with 1.4 M NaCl. The elute (about 2 ml) was added to a Millipore 10-kDa cut-off membrane, and 13 ml of Buffer C (20 mM Tris-HCl; 50 mM NaCl; 5 mM Na_2_EDTA; pH 7.5) was added, followed by centrifuging at 5000×g to the final volume of 1 ml. This step was repeated twice.

### Analyzing the enzymatic activity of the recombinant Endo H

The hydrolytic activity of Endo H from *P*. *pastoris* and commercial source (heterlogously expressed in *E*. *coli*) was compared with carboxypeptidase Y (CPY) and human erythropoietin (EPO) as substrates. About 10 U of Purified recombinant Endo H or commercial Endo H was added to 10 μg of CPY (Sigma, USA) and 10 μg of EPO (Cusabio, China), and incubated in proper buffer at 37°C for 1 h. Meanwhile, 10 U of PNGase F (NEB, USA) was used to deglycosylate EPO under the same condition. All samples were loaded for SDS-PAGE.

RNase B (10 μg) was denatured with 1× Glycoprotein Denaturing Buffer (0.5% SDS, 40 mM DTT) at 100°C for 10 minutes. After this treatment, 1 μl of reaction buffer (50 mM sodium citrate, pH 5.5) was added along with 1 μl of the dilutedrecombinant Endo H-P (5.0%, 4.5%, 4.0%, 3.5%, 3.0%, 2.5% and 2.0%); dH_2_O were added to the final volume of 10 μl. The mixture was incubated at 37°C for 1 h. SDS-PAGE was used to detect the hydrolyzed products. One unit (U) of Endo H was defined as the amount of enzyme required to remove >95% of carbohydrates from 10 μg of denatured RNase B in 1 h at 37°C in a total reaction volume of 10 μl (10NEB units = 1 IUB milliunit). The optimum temperature was determined by incubating 10 μg denatured RNase B with 1 μl reaction buffer and in a final volume of 10 μl at the proper temperature for 1 h. The optimum pH was determined by incubating 10 μg denatured RNase B with 1 μl reaction buffer adjusted to a series of pH values from 5.0 to 7.5 in a final volume of 10 μl at 37°C for 1 h. The experiments were performed in triplicate.

### Construction and enzyme activity of the substrates

Recombinant *P*. *pastoris* strains expressing endo-1, 4-β-mannosidase from *Aspergillus niger*, DNase I from *Bos taurus*, phytase from *E*. *coli* and *Dpn*I from *Diplococcus pneumoniae* were constructed in our lab. The recombinant strains expressing substrates were generated with the similar strategy as Endo H-P expressing *P*. *pastoris*. The coding sequences of these genes were optimized according to the codon usage preference of *P*. *pastoris* and synthesized with overlapping PCR. The newly synthesized ORFs were sequenced and inserted into pHBM905A vectors, followed by digesting with *Sal*I and transforming into *P*. *pastoris* GS115. The recombinants were screened on MD plates without histidine and identified with PCR [16]. The expressions of the foreign proteins were identified with SDS-PAGE.

### Deglycosylation of recombinant glycoproteins expressed in *P*. *pastoris* using the recombinant Endo H

For the post-fermentation treatment, the recombinant *P*. *pastoris* strains producing endo-1, 4-β-mannosidase, phytase and *Dpn*I were incubated in 100 ml BMGY until the OD_600_ value reached approximately 20. All cells were harvested by centrifugation, transferred to 25 ml BMMY and then induced with 1% (v/v) methanol for 144 h. The cell culture supernatant of the *P*. *pastoris* producing recombinant Endo H-P was prepared using the same procedure. The supernatant containing each glycoprotein was mixed with that containing recombinant Endo H-P at a ratio of 10:1 and incubated at 37°C for 1 h.

For co-fermentation, the recombinant *P*. *pastoris* strains expressing DNase I and phytase were separately inoculated in 100 ml BMGY and incubated until the OD_600_ value reached approximately 20. All cells were collected from these two samples and transferred into two flasks containing 25 ml BMMY in each. Cells from a 10-ml culture of recombinant Endo H-P-expressing *P*. *pastoris* prepared under the same conditions were collected and added to both flasks, which were then induced with 1% (v/v) methanol for 144 h.

### The growth curve of the recombinant strains under the co-fermentation condition

The effect of Endo H-P to the growth rate of the recombinant *P*. *pastoris* GS115 expressing phytase of *E*. *coli* was studied. *P*. *pastoris* strains expressing Endo H-P and phytase and *P*. *pastoris* GS115 strain bearing pHBM905A plasmid were incubated in 10 ml of BMGY separately until the OD_600_ reached approximately 1.0, then 1ml of the cultures were injected into 100 ml of BMGY separately. Meanwhile, 100-μl culture of the recombinant Endo H-P-expressing *P*. *pastoris* and 900-μl culture of the recombinant phytase-expressing *P*. *pastoris* were mixed and transferred into 100 ml of BMGY. OD_600_ of all four cell cultures were measured every 2 h. When OD_600_ of the cell cultures reached about 20, all cells were harvested by centrifugation and transferred to 50 ml BMMY. All four cell cultures were induced with 1% (v/v) methanol and OD _600_ was measured every 6 h.

### Deglycosylation of glycoproteins with Endo H-Pexpressed by *P*. *pastoris*

Glycoprotein staining was performed using the Thermo Scientific Pierce Glycoprotein Staining Kit in accordance with the manufacturer’s recommendations (USA). An equal amount of each sample was loaded onto two 15% (w/v) oligosaccharide gels. After SDS-PAGE, one gel was stained with Coomassie Brilliant Blue G-250, and the other was stained using the Glycoprotein Staining Kit.

### Analysis of the enzymatic activity of phytase and endo-1, 4-β-mannosidase after deglycosylation

The deoxyribonuclease activity of DNase I was analyzed by mixing 2 μl of pHBM905A (about 300 ng) with 0.5 μl of 10×DNase I Buffer and 1.5 μl of dH_2_O, followed by adding 1 μl of DNase I, and then incubated at 37°C for 1 min. The sample was treated at 75°C for 20 min before the agarose gel electrophoresis. The activity of endo-1, 4-β-mannosidase was analyzed using MD plates supplemented with 1% konjac powder and 0.05% trypan blue [20].

## Results

### Optimization and synthesis of the *Endo H* gene for the expression in *P*. *pastoris*

**Fig 1:**
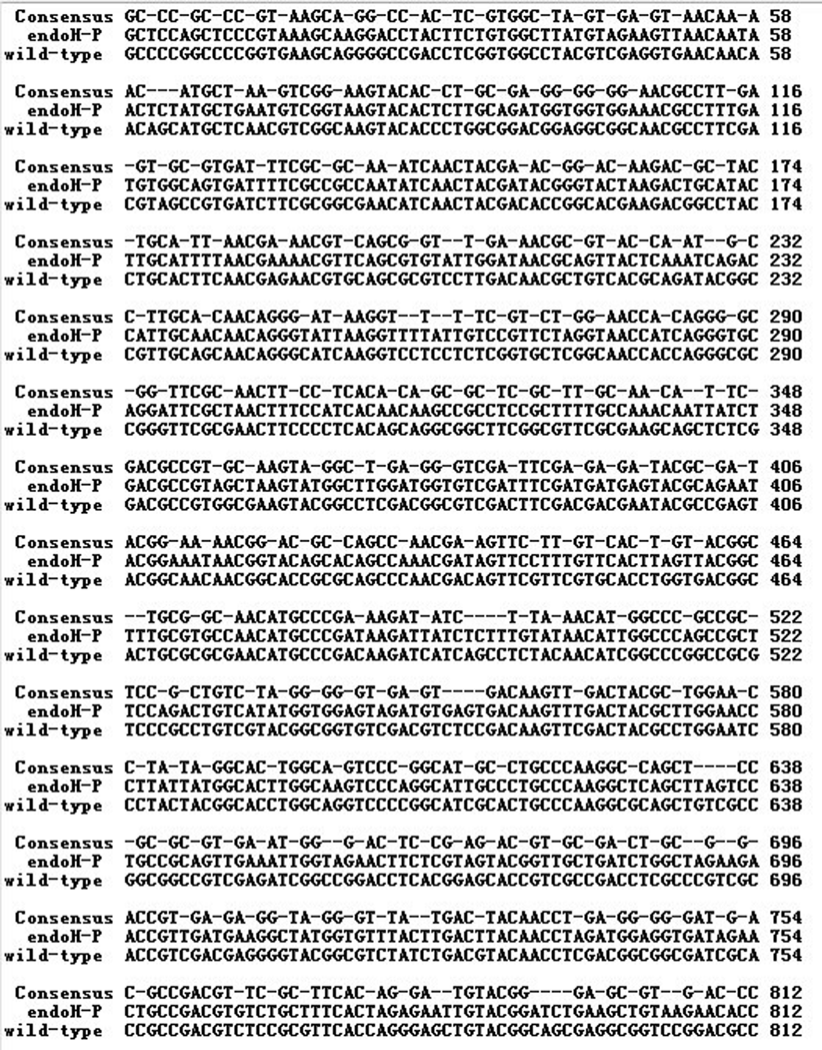
The alignments of *Endo H-P* with wild-type *Endo H*. The nucleotide sequence of *Endo* H-*P* was compared with that of wild-type *Endo* H, the result indicated that 220 nucleotides were altered in *Endo H-P*.

The *Endo H* gene of *S*. *plicatus* is 919 bp (GenBank accession AAA26738.1) and encodes a 28.9-kDa protein. The coding region, apart from the signal peptide (816 bp), was modified based on the codon usage bias of *P*. *pastoris* and synthesized by overlapping PCR. The new gene is hereafter referred to as *Endo H-P*. The alignment of *Endo H-P* with wild-type *Endo* H indicated that 220 nucleotides were altered in the synthesized ORF (Fig. 1). The *Endo H-P* fragment was cloned into a pHBM905A vector and fused with the MF-α leader sequence. The recombinant plasmid was termed pHBM-*Endo H-P*.

### Expression and identification of the recombinant Endo H

**Fig 2:**
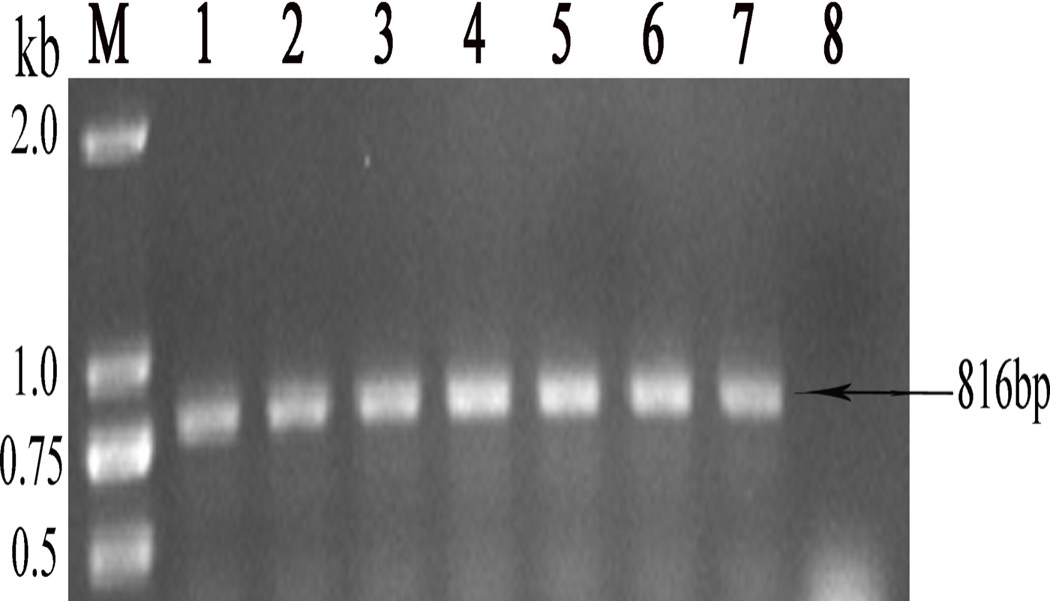
Whole-cell PCR to identify the recombinant *P*. *pastoris* bearing *Endo H-P* ORF. M DNA molecular weight markers (the size of each band was indicated on the left);Lane 1–6 PCR using 6 transformants as the templates; Lane 7 PCR with pHBM-*Endo H-P* plasmid as the template (positive control); Lane 8 PCR using *P*. *pastoris* bearing pHBM905A plasmid as the template (negative control).

The pHBM-*Endo H-P* plasmid was transformed into *P*. *pastoris* GS115. Six transformants were selected randomly from the MD plates and identified by whole-cell PCR using the primers EndoH-1 and EndoH-26 (Table 1). The results indicated that the target gene integrated into the chromosome through homologous recombination in all transformants (Fig. 2).

**Fig 3:**
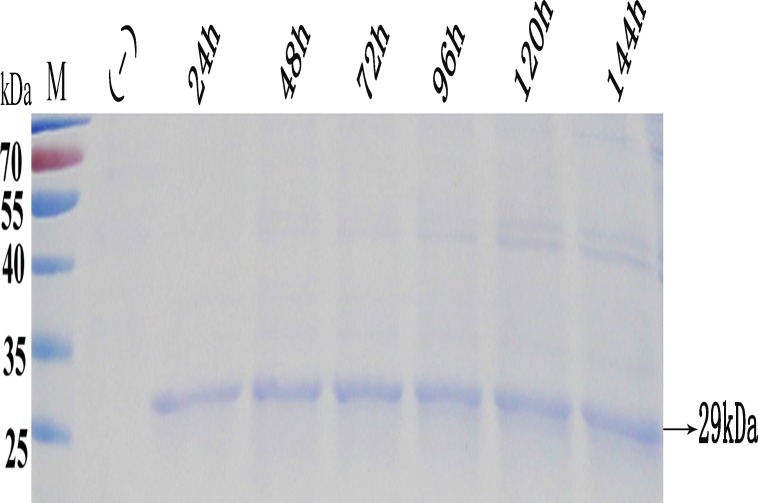
SDS-PAGE analysis of Endo H-P secreted in the cell culture supernatant of the shake flasks. M protein molecular weight marker (the size of each band was indicated on the left); Lane 1 cell culture supernatant (15 μl) of the strain bearing pHBM905A plasmid (negative control); Lane 2–7 cell culture supernatants (15 μl) collected from 1 to 6 days, respectively.

One transformant was selected randomly for the expression of Endo H-P. SDS-PAGE of the supernatants showed the presence of an approximately 29-kDa main band from the first day of induction (Fig. 3, the position of the target band is indicated by an arrow). The total protein in the supernatant was 259, 288, 292 and 380 mg/l from day 1 to day 4, respectively. The expression of the recombinant Endo H reached a maximum level at 120 h, with a protein concentration of approximately 397 mg/l. And then, the concentration of the target protein decreased to 373 mg/l at 144 h. These results demonstrated the successful expression of Endo H in *P*. *pastoris*. The recombinant protein was termed Endo H-P.

### Purification and activity assay of recombinant Endo H-P

**Fig 4:**
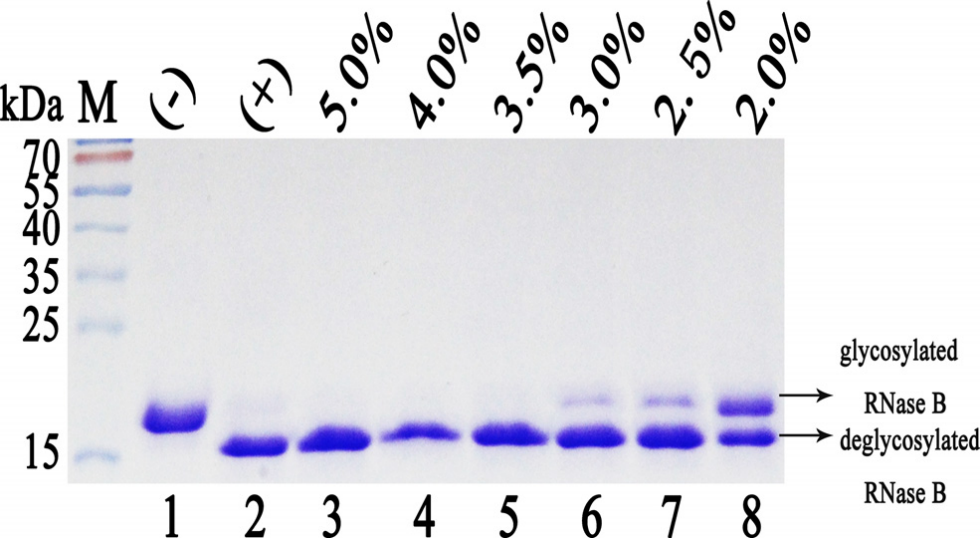
Determining the enzyme activity of Endo H-P through mobility shift assay of RNase B. M protein molecular weight markers (the size of each band was indicated on the left); Lane 1 denatured RNase B (negative control); Lane 2 denatured RNase B treated with 1 U of commercial Endo H from NEB, USA (positive control); Lane 3–8 denatured RNase B treated with 1μl of Endo H-P diluted into 5.0%, 4.0%, 3.5%, 3.0%, 2.5% and 2.0%, respectively.

The fermentation supernatant was purified and the protein concentration was 61.9 mg/l after purification. Enzymatic activity was measured using denatured RNase B as the substrate. Endo H digestion results in the release of the glycan side-chains from RNase B, yielding a decrease in molecular weight from 17 kDa to approximately 15 kDa and a corresponding mobility shift during SDS-PAGE. As a control, 1U commercial Endo H (NEB, USA) was found to remove almost all of the carbohydrates from the denatured RNase B following incubation at 37°C for 1 h (Fig. 4). At least 1 μl of 3.5%-fold-diluted Endo H-P was required to achieve the same level of digestion (Fig. 4). Accordingly, the enzymatic activity of Endo H-P was determined to be approximately 461573 U/mg.

**Fig 5:**
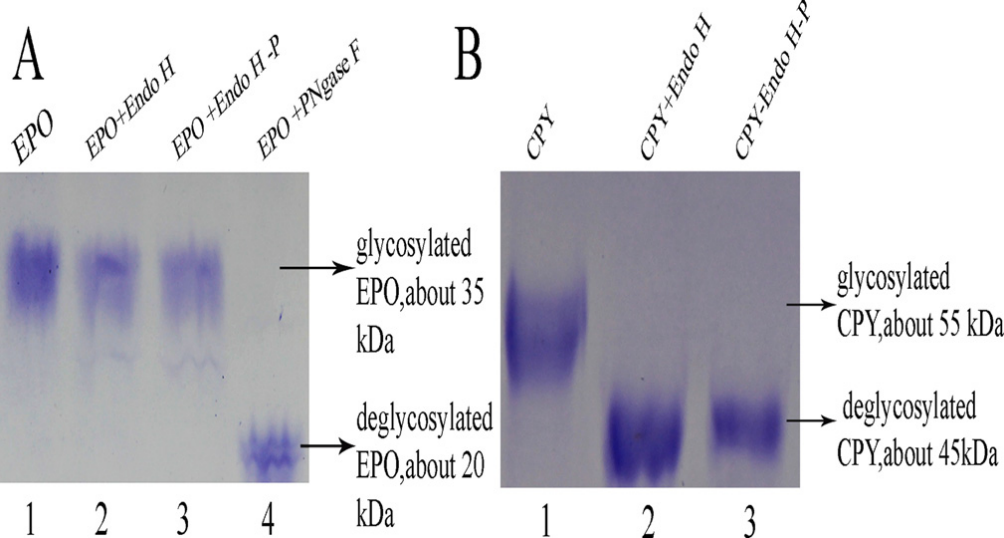
The hydrolytic activity of Endo H-P to proteins from mammals and baker's yeast. (A). The hydrolytic activity of Endo H-P to EPO expressed in CHO cells. Lane 1 EPO without treatment; Lane 2 EPO treated with commercial Endo H; Lane 3 EPO treated with purified Endo H-P; Lane 4 EPO treated with commercial PNGase F. (B). The hydrolytic activity of Endo H-P to CPY from baker's yeast. Lane 1 CPY without treatment; Lane 2 CPY treated with commercial Endo H; Lane 3 CPY treated with purified Endo H-P.

Meanwhile, the glycohydrolase activity of Endo H-P to other substrates from mammals and Baker's yeast was compared with recombinant Endo H from *E*. *coli*. Both of them could remove N-linked glycans of CPY and RNase B but not EPO, which was able to be cleaved by PNGase F (Fig. 5). This result indicated that Endo H-P expressed in different system was able to deglycosylate proteins from mammals, and the sensitivity was various to different glycoproteins.

### Characteristics of Endo H-P

**Fig 6:**
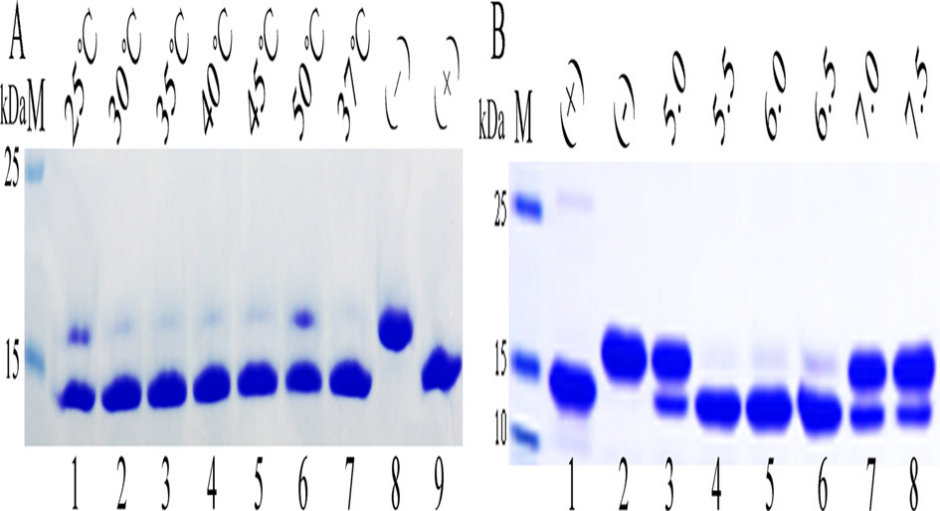
Analyzing the characteristics of Endo H-P through mobility shift assay of RNase B. (A).Identifying the optimum temperature of Endo H-P with SDS-PAGE. M protein molecular weight markers (the size of each band was indicated on the left);Lane 1 to Lane 6 denatured RNase B treated with concentrated Endo H-P at 25°C, 30°C, 35°C, 40°C, 45°C and 50°C, respectively; Lane 7 denatured RNase B treated with concentrated Endo H-P at 37°C, respectively;Lane 8 the negative control (RNase B without treatment); Lane 9 the positive control (overdose of Endo H-P was added to the reaction system);(B). Identifying the optimum temperature of Endo H-P with SDS-PAGE. M protein molecular weight markers (the size of each band was indicated on the left);Lane 1 the positive control (overdose of Endo H-P was added to the reaction system); Lane 2 the negative control (RNase B without treatment);Lane 3–8 denatured RNase B treated with Endo H-P at pH5.0, 5.5, 6.0, 6.5, 7.0 and 7.5, respectively.

Endo H-P efficiently cleaved the N-glycan side-chains from RNase B at temperatures ranging from 30°C to 45°C, and 37°C was determined to be the optimum temperature (Fig. 6A). EndoH-P showed the highest activity at pH 5.5 to 6.5, and the optimum pH was 5.5 (Fig. 6B). These results were consistent with those obtained using commercial Endo H.

### Deglycosylation of recombinant proteins expressed in *P*. *pastoris* by post-fermentation treatment

**Fig 7:**
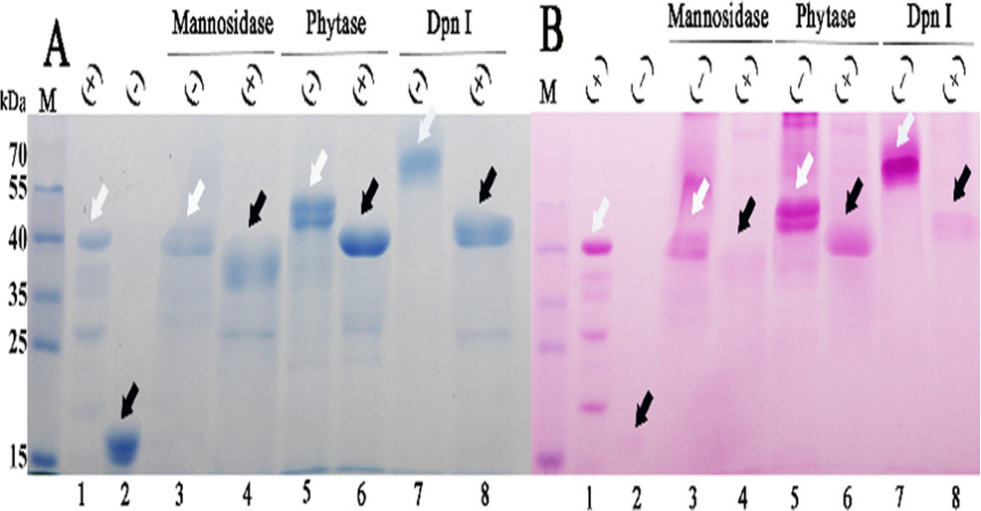
Deglycosylation of recombinant proteins expressed in *P*. *pastoris*. (A). SDS-PAGE of the recombinant proteins expressed in *P*. *pastoris* with or without digestion of Endo H-P with post-fermentation method. M protein molecular weight markers (the size of each band was indicated on the left); Lane 1 positive control(Horseradish Peroxidase) from the Glycoprotein Staining Kit;Lane 2 negative control(Soybean Trypsin Inhibitor) from Glycoprotein Staining Kit;Lane 3 mannosidase expressed in *P*. *pastoris*;Lane 4 deglycosylated mannosidase; Lane 5 phytase expressed in *P*. *pastoris*; Lane 6 deglycosylated phytase;Lane 7 *Dpn*I expressed in *P*. *pastoris*;Lane 8 deglycosylated *Dpn*I. (B). Glycoprotein staining of the recombinant proteins expressed in *P*. *pastoris* with or without Endo H-P digestion. All samples were loaded in the identical order with A.

The fermentation supernatant containing Endo H-P was mixed with the fermentation supernatants from *P*. *pastoris* cultures expressing endo-1, 4-β-mannosidase, phytase and *Dpn*I. Glycosylation staining indicated that these recombinant proteins were strongly glycosylated (Fig. 7B), and the sizes of these enzymes decreased significantly following Endo H-P treatment (Fig. 7A, the bands of the target proteins are indicated by open arrows, and the bands corresponding to deglycosylated proteins are shown by black arrows), as did the glycosylation staining intensities (Fig. 7B). These results demonstrate that the fermentation supernatant of Endo H-P may be used to directly deglycosylate proteins heterologously expressedin eukaryotic cells, thereby facilitating the purification of deglycosylated proteins. In addition, trace glycosylation staining was still detected in the recombinant proteins after digestion, which indicated that these recombinant proteins also underwent O-linked glycosylation.

### Deglycosylation of recombinant proteins through co-fermentation of two recombinant *P*. *pastoris* strains

**Fig 8:**
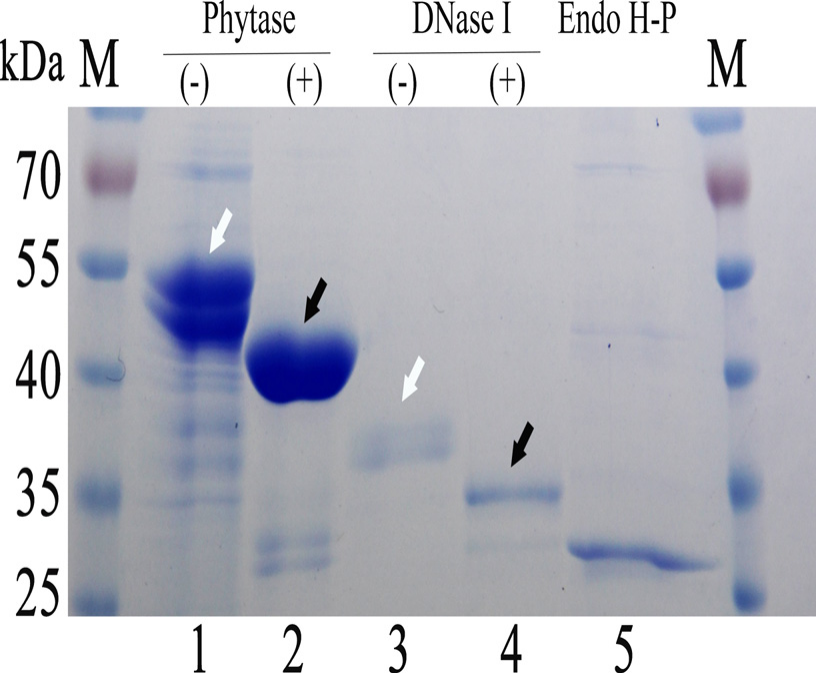
Co-fermentaion of EndoH-P with the recombiant DNase I and phytase. M protein molecular weight markers (the size of each band was indicated on the left); Lane 1 phytase expressed in *P*. *pastoris*; Lane 2 phytase co-induced with Endo H-P in *P*. *pastoris*; Lane 3 DNase I expressed in *P*. *pastoris*; Lane 4 DNase I co-induced with Endo H-P in *P*. *pastoris*; Lane 5 Endo H-P expressed in *P*. *pastoris*.

The predicted sizes of *E*. *coli* phytase and *Bos Taurus* DNase I were 47 kDa and 31 kDa, respectively, whereas upon expression in *P*. *pastoris*, the observedsizes of these two proteins were much bigger due to glycosylation (Fig. 8, open arrows). The apparent sizesdecreased to match the predicted sizes after co-fermentation with the Endo H-P-expressing strain in shake flasks (Fig. 8, black arrows). Moreover, two main bands in close proximity were detected for the recombiant phytase, and only one smaller band was observed in the corresponding co-fermentation sample. These results were consistent with the post-fermenation treatment, indicating that both the post-fermentation and co-fermentaion treatments remove glycan chains in the same manner.

**Fig 9:**
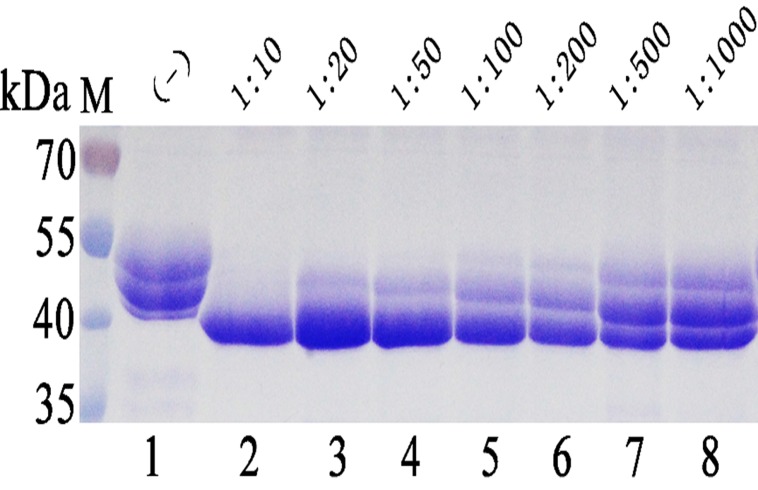
Co-fermentaion the phytase expressing *P*. *pastoris* and Endo H-P expressing *P*. *pastoris* with various initial ratio of cells innoculated in BMMY medium. M protein molecular weight markers (the size of each band was indicated on the left); Lane 1 phytase expressed in *P*. *pastoris*; Lane 2–8 EndoH-P-expressing *P*. *pastoris* co-fermentated with phytase-expressing *P*. *pastoris* with inoculation ratio of 1:10, 1:25, 1:50, 1:100, 1:200, 1:500 and 1:1000 (Endo H-P: phytase), respectively.

Meanwhile, the Endo H-P-expressing strain and phytase-expressing strain were mixed with different ratio in shake flasks. The result indicated that 10% of Endo H-P expressing cells was the minimal amount need to completely remove the N-glycan side-chains of phytase (Fig. 9).

### The effect of deglycosylation to the growth of the phytase-expressing *P*. *pastoris* strain

**Fig 10:**
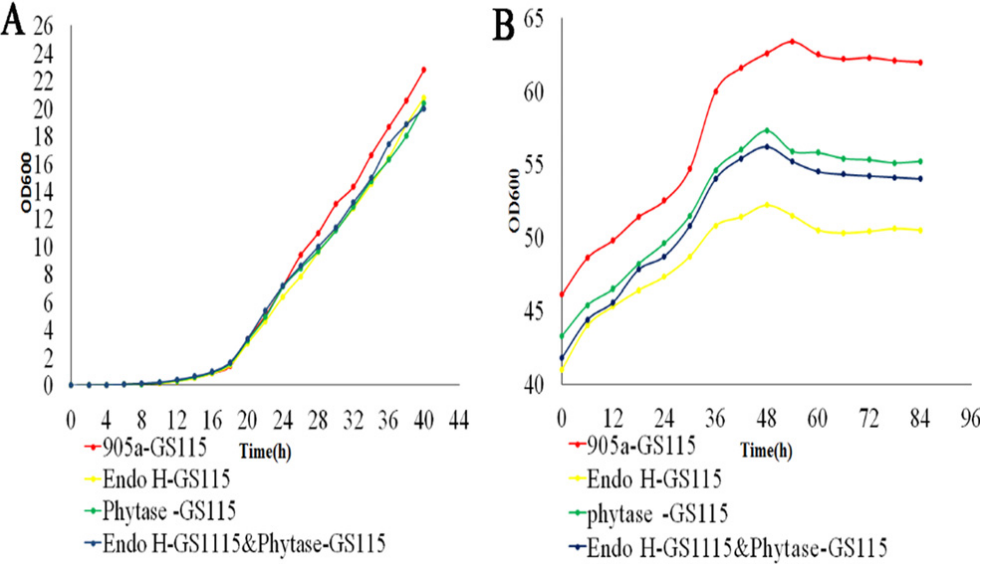
The growth curves of phytase expressing *P*. *pastoris* with or without co-fermentation with Endo H-P expressing *P*. *pastoris*. (A).The growth curve of phytase- and Endo H-P-expressing *P*. *pastoris* during the vegetative growth phase. (B). The growth curve of phytase- and Endo H-P- expressing *P*. *pastoris* during the induction phase. The names of the strains were indicated at the bottom of the panel.

The growth rate of phytase-expressing strain with or without co-fermentation with Endo H-P was analyzed. Duing the vegetative phase, the phytase- and Endo H-P-expressing strain grew under similar speed with *P*. *pastoris* GS115 strain bearing pHBM905A, and OD_600_ of the co-fermention sample increased at the same trend (Fig. 10 A). When induced with methenol, both Endo H-P- and phytase-expressing strain grew slower than the *P*. *pastoris* GS115 strain bearing pHBM905A. The co-fermention sample grew at the simliar speed as phytase-expressing strain (Fig. 10 B). These result indicated that deglycosylation of phytase had no obvious effect to the growth of recombinant strain.

### Analysis of the enzymatic activity of phytase and endo-1, 4-β-mannosidase after deglycosylation

**Fig 11:**
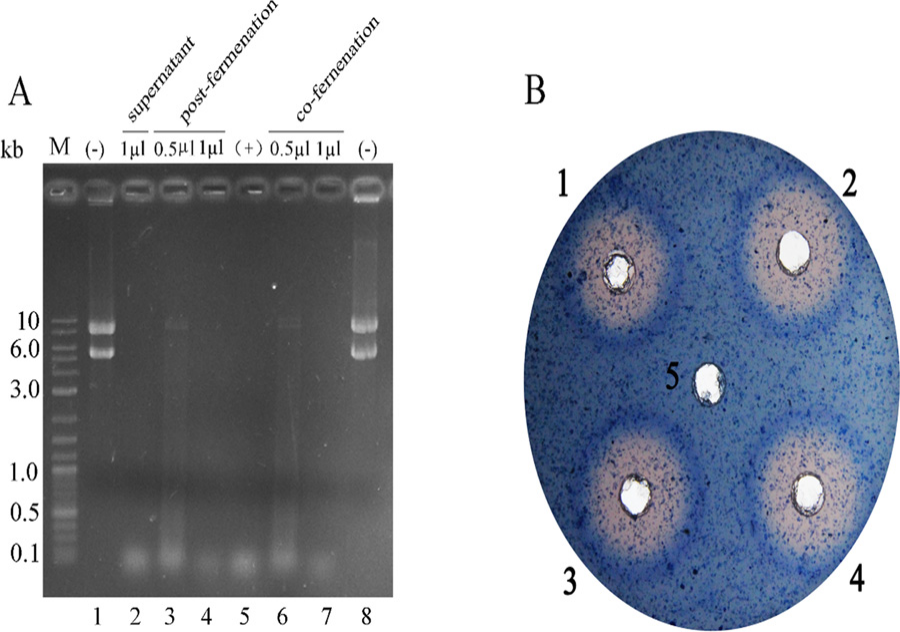
Analysis of the enzymatic activity of deglycosylated DNase I and endo-1, 4-β-mannosidase obtained from co- and post-fermentation with Endo H-P. (A). M DNA molecular weight markers (the size of each band was indicated on the left); Lane 1 pHBM905A plasmid (about 300 ng); Lane 2 pHBM905A treated with 1 μl supernatant of DNase I; Lane 3–4 pHBM905A treated with 0.5 and 1 μl of deglycosylated DNase I with post-fermentation treatment; Lane 5 pHBM905A treated with 1 U commercial DNase I; Lane 6–7 pHBM905A treated with 0.5 and 1 μl of deglycosylated DNase I with co-fermentation treatment; Lane 8 pHBM905A treated with fermentation supernatant from *P*. *pastoris* bearing pHM905A plasmid (the negative control). (B). A hole of about 2mm was made with a hole puncher on a MD plate supplemented with 1% konjac powder and 0.05% trypan blue. The samples were added into the wells and the plate was incubated at 37°C, overnight. Sample 1: 1.5 μl supernatant of *P*. *pastoris* expressing glycosylated endo-1, 4-β-mannosidase; Sample 2: 2 μl supernatant of *P*. *pastoris* expressing glycosylated mannanase; Sample 3: 2 μl supernatant of deglycosylated endo-1, 4-β-mannosidase with post-fermentation treatment; Sample 4: 2 μl supernatant of deglycosylated endo-1, 4-β-mannosidase with co-fermentation treatment; Sample 5: 2 μl supernatant of EndoH-P.

No DNA band can be detected after pHBM905A plasmid was treated with DNase I of the glycosylated form or deglycosylated form obtained from co- or post-fermentation(Fig. 11 A). Meanwhile, the konjac powder in the plate was degraded by the endo-1, 4-β-mannosidase with the same treatment and a clear halo could be detected around the samples (Fig. 11 B). These results indicated that both enzymes still remained active after deglycosylation.

## Discussion

Glycosylation is a common post-translational modification in eukaryotic cells. When expressed in different eukaryotic expression systems, the same gene of interest can yield products of various molecular weights due to the differing glycosylation patterns of the recombinant proteins. For example, in *S*. *cerevisiae*, recombinant human erythropoietin (EPO) has a molecular weight above 29 kDa [21], whereas it is approximately 35 kDa and 25 kDa in CHO cells [22] and *Drosophila* S2 cells [23], respectively. Because glycosylation is closely linked to the folding, stability and activity of proteins, abnormal modifications may affect protein function. *P*. *pastoris* which is a commonly used eukaryotic host for heterologous expression, has a complex glycosylation system [24], and although previous reports have indicated that over-glycosylation may enhance the thermodynamic and kinetic stability of recombinant proteins [25], some studies have indicated that the hyper-glycosylation that occurs in *P*. *pastoris* may compromise function [26–27]. For example, the native Pfs48/45 protein does not contain N-linked glycans; however, following expression in *P*. *pastoris*, it loses its transmission-blocking activity and does not induce transmission-blocking antibodies in mice due to the modification of some potential recognition sites by the *P*. *pastoris* glycosylation system [28]. Therefore, glycohydrolases are not only important for identifying the composition of the glycan portion of glycoproteins but are also useful for preparing active enzymes Endo H is particularly important among glycohydrolases. In this study, we expressed high levels of Endo H using a *P*. *pastoris* expression system and investigated the application of this system in the deglycosylation of glycoproteins. The expression level of the target protein in the supernatant of the shake flasks was 397 mg/l and 61.9 mg/l after purification, which was 20-fold higher than the yield in *E*. *coli* and silkworm. The recombinant Endo H from *P*. *pastoris* and *E*. *coli* could remove N-linked glycan from mammal and Baker's yeast glycoproteins, such as RNase B and CPY. And it could also cleave polysaccharides from glycoproteins heterolgously expressed in *P*. *pastoris*. In addition, it reported that recombinant Endo H from silkworm-BES was able to cleave the N-glycan from RNase B and the high-mannose glycoproteins from silkworm hemolymph [15]. The results implied that Endo H could work to glycoproteins from various eukaryotic organisms, even though mammals and yeasts have very different post-translational modification pathway [29–30]

The process of purification is labor consuming and most target proteins were lost during the purification. Therefore, we explored simple methods to apply Endo H-P. Co-expressing bacterial PNGase F with Pfs48F1 in plant cells was found to cause the deglycosylation of this malaria vaccine candidate, which in turn led to stronger recognition by several antibodies compared with the glycosylated form [31–32]. Hence, the *in vivo* deglycosylation of proteins expressed in eukaryotic cells has practical uses. Although this method facilitated the process of deglycosylation, co-expression may cause competition for resources used in protein synthesis and thus decrease target protein yields. The recombinant Endo H expressed in this study was approximately 29 kDa, which was close to the calculated size. According to our previous studies, most enzymes expressed in *P*. *pastoris* are larger than their predicted sizes due to glycosylation [33]. We deduced that the glycan side chains of Endo H-P may have been self-cleaved in culture, implying that this recombinant glycohydrolase may have been sufficiently active in the fermentation supernatant. Therefore, we constructed a *P*. *pastoris* strain to independently express high levels of Endo H. This strain was used to deglycosylate several recombinant proteins expressed in our lab using co-fermentation and post-fermentation methods. The results indicated that the Endo H-P secreted by this strain can specifically and efficiently catalyze deglycosylation reactions via both methods, thereby simplifying the process of deglycosylation. In addition, it can also avoid the loss of Endo H-P during the process of purification and saving the cost of the complicated purification. Moreover, the ratio of glycoproteins to Endo H can be adjusted more flexibly using this genetically engineered strain, which in turn, could grantee the N- linked glycan to be fully cleaved.

In summary, we have successfully expressed Endo H using a *P*. *pastoris* expression system and achieved expression levels of 397 mg/l with the specific activity of about 461573 U/mg. The N-linked glycan side-chains of several recombinant proteins can be conveniently removed using this glycohydrolase. Future studies will involve the co-expression of glycohydrolases that can remove both O-linked and N-linked glycan side-chains to obtain completely deglycosylated substrates.
