# Correction: High-Level Expression of Endo-β-N-Acetylglucosaminidase H from *Streptomyces plicatus* in *Pichia pastoris* and Its Application for the Deglycosylation of Glycoproteins

**DOI:** 10.1371/journal.pone.0137929

**Published:** 2015-09-03

**Authors:** Fei Wang, Xiaojuan Wang, Xiaolan Yu, Ling Fu, Yunyun Liu, Lixin Ma, Chao Zhai

The fourth author’s name is spelled incorrectly. The correct name is: Ling Fu.
